# Genomic Surveillance of a Globally Circulating Distinct Group W Clonal Complex 11 Meningococcal Variant, New Zealand, 2013–2018

**DOI:** 10.3201/eid2704.191716

**Published:** 2021-04

**Authors:** Zuyu Yang, Xiaoyun Ren, Heather Davies, Timothy Wood, Liza Lopez, Jill Sherwood, Audrey Tiong, Philip E. Carter

**Affiliations:** Institute of Environmental Science and Research, Porirua, New Zealand

**Keywords:** meningococcal disease, whole-genome sequencing, group W, evolution, Public Health Surveillance, New Zealand, bacteria, meningitis/encephalitis, invasive meningococcal disease, *Neisseria meningitidis*

## Abstract

Genomic surveillance is an essential part of effective disease control, enabling identification of emerging and expanding strains and monitoring of subsequent interventions. Whole-genome sequencing was used to analyze the genomic diversity of all *Neisseria meningitidis* isolates submitted to the New Zealand Meningococcal Reference Laboratory during 2013–2018. Of the 347 isolates submitted for whole-genome sequencing, we identified 68 sequence types belonging to 18 clonal complexes (CC). The predominant CC was CC41/44; next in predominance was CC11. Comparison of the 45 New Zealand group W CC11 isolates with worldwide representatives of group W CC11 isolates revealed that the original UK strain, the 2013 UK strain, and a distinctive variant (the 2015 strain) were causing invasive group W meningococcal disease in New Zealand. The 2015 strain also demonstrated increased resistance to penicillin and has been circulating in Canada and several countries in Europe, highlighting that close monitoring is needed to prevent future outbreaks around the world.

*Neisseria meningitidis*, a gram-negative bacterium, is the causative agent for meningococcal meningitis and septicemia and has been associated with isolated cases, outbreaks, and epidemics worldwide (*1*). The rapid progression of invasive meningococcal disease (IMD) and its high incidence of severe illness and death make IMD a feared and closely monitored disease. *N. meningitidis* is classified into 12 groups on the basis of capsular polysaccharide structure, but 6 groups (A, B, C, W, X, and Y) cause most life-threatening IMD (*2*). Most meningococcal disease is caused by hyperinvasive lineages belonging to specific clonal complexes (CC) as defined by multilocus sequence typing (MLST) (*3*), including CC32, CC41/44, CC11, and CC5 (*4*). Obtaining complete genetic information and resolving the evolutionary relationships of invasive pathogens are vital for identifying the origins and expansion of new pathogenic strains.

Group W CC11 (W:CC11) meningococci emerged as a global cause of IMD after an outbreak in Mecca, Saudi Arabia, in 2000 (*5*). High levels of group W disease have recently occurred in many countries and regions, including the United Kingdom (*6*), Sweden (*7*), Australia (*8*), and North America (*9*,*10*), and whole-genome sequencing (WGS) has been used to investigate its spread. Core-genome MLST found that group W:CC11 isolates were distinct from groups B and C sequence type (ST) 11 isolates (*11*). Currently, 4 major W:CC11 strains belonging to 2 major lineages are circulating globally: the Hajj strain sublineage, including the Hajj strain (*12*), and the South America strain sublineage, which includes the South America strain that emerged in 2003 in southern Brazil (*13*); the UK strain, a variant of the South America strain; and the 2013 UK strain (*14*), which has expanded into several countries (*8*,*9*,*15*).

During 1991–2008, New Zealand experienced a prolonged epidemic of IMD; most cases were caused by a single *N. meningitidis* group B strain (NZMenB), defined by PorA type P1.7–2,4 and belonging to CC41/44 (*16*,*17*). A strain-specific vaccine, MeNZB, was introduced in 2004 (*18*) and withdrawn in 2008 after rates of IMD decreased (*19*). The NZMenB strain continues to cause about one third of meningococcal infections in New Zealand, but other group B, C, W, and Y strains are also circulating (*20*). Recently, incidence of IMD caused by W:CC11 has increased in New Zealand. To learn more about the genetic diversity associated with IMD after the epidemic, we used WGS to analyze 347 isolates collected during 2013–2018. We determined their clonal relationship and compared the genomes of the New Zealand W:CC11 isolates with global representatives of W:CC11 lineages. 

## Methods

### Surveillance and Epidemiologic Analysis

Meningococcal disease is a notifiable disease in New Zealand; all IMD cases are referred to the Meningococcal Reference Laboratory at the Institute of Environmental Science and Research (ESR) for routine grouping using slide agglutination or PCR (*21*). For this study, disease incidence and case demographics were derived from notification data extracted from the national notifiable disease surveillance database. Annual population denominators were taken from Statistics New Zealand. We used R version 3.4.4 (https://www.r-project.org) to perform all statistical tests. Shannon–Wiener diversity index was calculated by using vegan version 2.5.6 (*22*).

### WGS

We analyzed all available meningococcal isolates from 2013–2018 in New Zealand by WGS (Appendix 1 Table 1). Genomic DNA was purified by using the Gentra Puregene Yeast/Bact. Kit (QIAGEN, https://www.qiagen.com) or High Pure PCR Template Preparation Kit (Roche, https://www.roche.com) according to the manufacturer’s protocols. A 10-µL loop of bacteria, grown overnight on Columbia blood agar plates (Fort Richard Laboratories, Auckland, NZ), was suspended in 300 µL of lysis buffer and heat-killed at 56°C for 1 h. We then quantified DNA by using the Quant-iT PicoGreen dsDNA assay kit (Thermo Fisher Scientific, https://www.thermofisher.com) and constructed libraries by using Nextera-XT DNA Library Preparation Kit (Illumina, https://www.illumina.com). Paired-end sequencing of 2 × 150 bp was performed on the Illumina platform at ESR. Read data are available for download from the National Center for Biotechnology Information Sequence Read Archive (Bioproject accession no. PRJNA592848) and from PubMLST (https://pubmlst.org).

### Genomic Analysis

Raw reads were quality trimmed by using Trimmomatic version 0.32 (*23*) to remove adapters, low quality bases (<Q20), and reads shorter than 60 bp. SPAdes version 3.10.1 (*24*) was used for assembly, and contigs >200 bp were kept. Assembled contigs were used for in silico MLST, capsular grouping, and antigen typing by using meningotype version 0.82-β (*25*). Single-nucleotide polymorphisms (SNPs) were identified by aligning quality-trimmed paired-end reads to the *N. meningitidis* reference sequence FAM18 (GenBank accession no. AM421808.1) and using Bowtie2 version 2.2.5 (*26*). Alignments were processed with Picard-tools (*27*) to remove duplicated reads and assessed with Qualimap version 2.2.1 (*28*) (Appendix 1 Table 2). We used FreeBayes version 1.2.0–2-g29c4002 (E. Garrison, unpub. data, https://arxiv.org/abs/1207.3907) to detect sequence variations among isolates with these settings: ploidy = 1, at 20× minimum depth and 70% minimum variant allele frequency. We filtered variants by using vcflib (*29*) and vcftools version 0.1.12b (*30*) and removed sites located in the tandem repeat regions by using the intersect function from BEDTools version 2.23.0 (*31*). For datasets that included only assembled genomes, we used Parsnp version 1.1.2 (*32*) to perform core-genome alignment and FAM18 (AM421808.1) as reference.

### Phylogenetic Analysis

We constructed phylogenetic analyses from core SNP alignment by using the maximum-likelihood method under the general time reversible substitution model with RAxML version 8.2.12 (*33*) and estimated the relative robustness of the clades with 200 bootstrap replicates (*34*). We used ClonalFrameML version 1.25 (*35*) to construct recombination-corrected phylogeny on the basis of core SNP tree topology. For datasets that included only assembled genomes, we generated a maximum-likelihood phylogenetic tree by using RAxML on the basis of the core-genome alignment with 200 bootstrap replicates. Phylogenies were annotated by using iTOL (*36*).

### Selection of Global Group W:CC11 Isolates

We compared New Zealand group W:CC11 isolates with 153 short-read datasets and 30 draft assemblies. Short-read data in the European Nucleotide Archive were selected to represent all major lineages of group W:CC11. We followed studies by Lucidarme et al. (*14*) and Tsang et al. (*9*) and randomly chose 1–3 isolates per year per country. We selected 153 datasets including 151 group W isolates and 2 group C isolates (ERR557598 and ERR976806), which were used as an outgroup to study the genetic relationship within group W:CC11.

We used the PubMLST *Neisseria* database to identify other isolates closely related to the New Zealand ST11 isolates (*37*). As of February 18, 2019, a total of 30 isolates were within the 50 mismatch thresholds to NZ17MI0022 based on comparison of the *N. meningitidis* core-genome MLST (*38*). Draft assemblies of these 30 isolates were downloaded from PubMLST. Short-read data were available for 3 of these 30 isolates. We compiled detailed information on sequences used for phylogenetic analysis (Appendix 1 Table 3).

### Antimicrobial Susceptibility Testing of New Zealand Group W:CC11 Isolates

Of the 45 New Zealand invasive group W:CC11 isolates, 42 were assessed for susceptibility to penicillin, ciprofloxacin, ceftriaxone, and rifampin. MICs were determined by gradient strip on Mueller-Hinton agar with 5% sheep blood and interpreted according to the Clinical and Laboratory Standards Institute breakpoints (*39*). Assembled contigs were used for in silico penA typing by using the PubMLST *Neisseria* database.

## Results

### Epidemiology of Meningococcal Disease in New Zealand, 2013–2018

**Figure 1:**
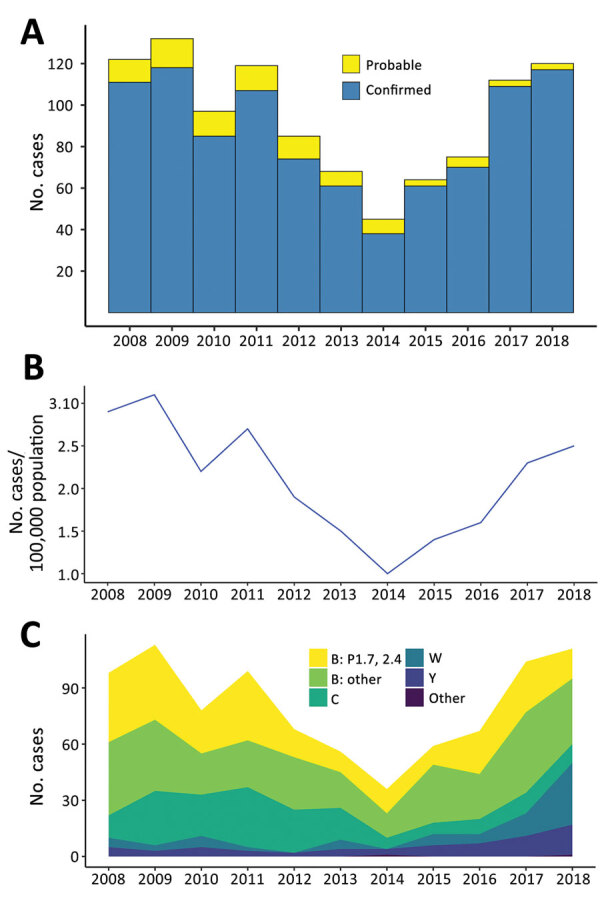
Epidemiology of meningococcal disease, New Zealand, 2013–2018. A) Number of confirmed and probable cases. B) Number of cases per 100,000 population of meningococcal disease. C) Proportion of meningococcal disease by group.

During 2013–2018, a total of 484 cases of meningococcal disease were reported, including 456 confirmed and 28 probable cases, for a confirmation rate of 94.2% (Figure 1, panel A; Appendix 1 Table 4). Rates of IMD (Figure 1, panel B) continued to fall after the end of the group B epidemic in 2008 (coefficient = −2.619, R^2^ = 0.6947; p = 0.0245) but increased during 2014–2018 (coefficient = 2.481, R^2^ = 0.9567; p = 0.0025). Inflection point of the time series was identified by breakpoint analysis and using the R package strucchange (*40*) and tested by the Chow test in R by using monthly disease rates (cases/100,000) during January 2008–December 2018 (Appendix 1 Table 5). The test identified the inflection point as October 2013 (95% CI January–December 2013).

Group B meningococci continue to be responsible for most IMD cases in New Zealand. Among the 438 confirmed cases analyzed by ESR during 2013–2018 were 265 (54.7%) group B (100 NZMenB and 165 other group B) cases, 58 (13.2%) group C cases, 61 (13.9%) group W cases, and 47 (10.7%) group Y cases (Figure 1, panel C; Appendix 1 Table 6). To compare whether significant changes occurred during 2013–2018, we used a 2-proportion Z-test in R to compare the proportion of diseases caused by each group. We found that the proportion of group W disease in 2018 (33/133) was significantly greater than that in 2013 (5/58, χ^2^ = 8.2415; p = 0.002047). In contrast, the proportion of disease caused by group C in 2018 (10/133) is significantly less than that of 2013 (17/58, χ^2^ = 10.578; p = 0.00057).

**Figure 2:**
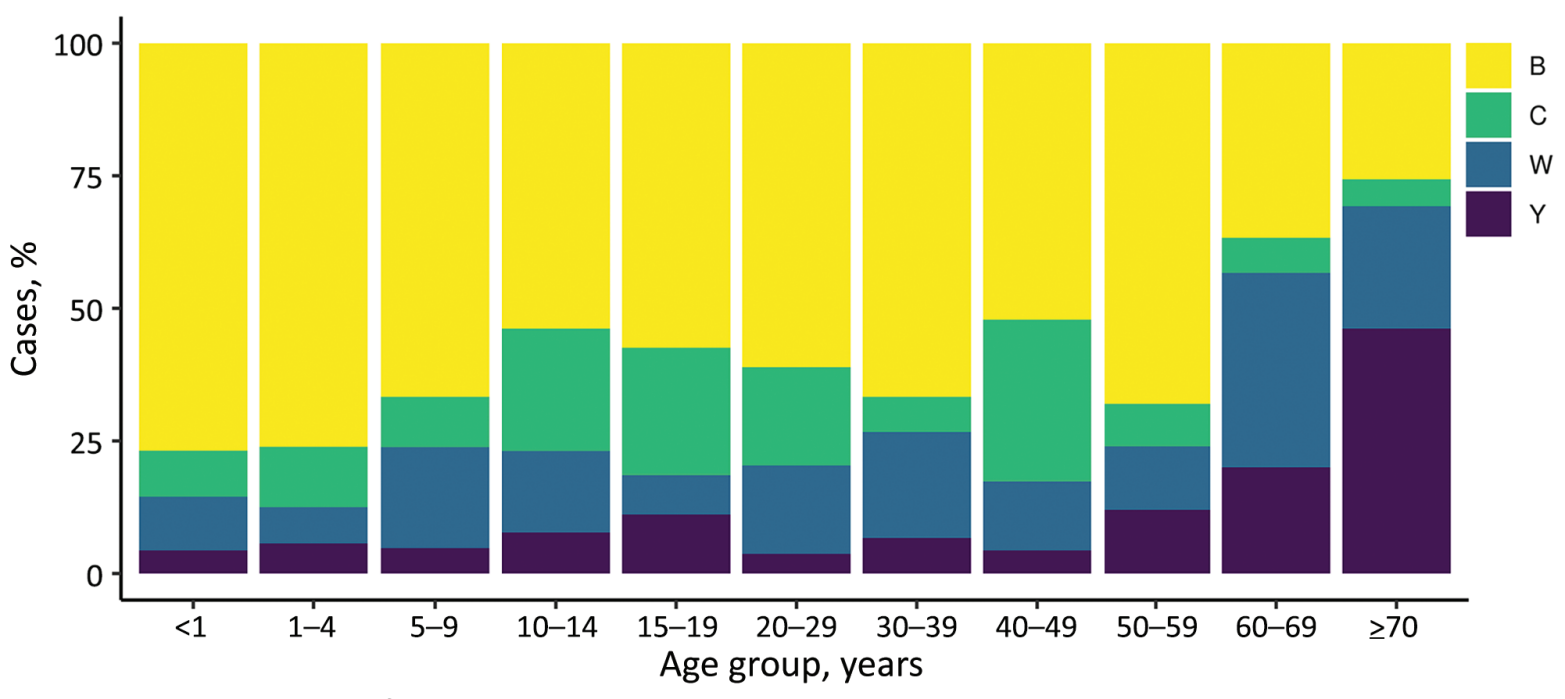
Age group distribution of meningococcal disease, by isolate group, New Zealand, 2013–2018

The rate of IMD continues to be highest among patients <1 year of age (from 10.2/100,000 population in 2014 to 23.1/100,000 population in 2017); rates are second highest among children 1–4 years of age (from 5.2/100,000 population in 2014 to 9.8/100,000 population in 2017). Adults >60 years of age are more affected by group W and Y disease (Figure 2; Appendix 1 Table 7).

### Clonal Distribution of Circulating Meningococci

We performed WGS and in silico typing on the 347 New Zealand meningococcal isolates (288 invasive and 59 noninvasive) collected during 2013–2018. The WGS dataset includes 175 group B isolates (65 NZMenB and 110 other group B), 48 group C isolates, 2 group E isolates, 64 group W isolates, 47 group Y isolates, 1 group X isolate, and 8 nongroupable isolates. MLST analysis showed that the 347 isolates contained 49 known STs. The 10 most common STs were ST11 (85), ST154 (43), ST23 (21), ST42 (17), ST32 (16), ST213 (15), ST1655 (15), ST1572 (11), ST6058 (8), and ST22 (7). There were 39 other STs with a single isolate and 19 unassigned STs (submitted through this project). The STs identified belonged to 18 clonal complexes. A total of 93 unique strains were defined by combination of group:PorA-variable region (VR)1, PorA-VR2:FetA:CC, with a Shannon–Wiener diversity index of 3.516. Bexsero antigen sequence types analysis showed 69 unique types with 23 isolates in which type could not be determined because of the absence of required loci (Appendix 1 Table 1).

**Figure 3:**
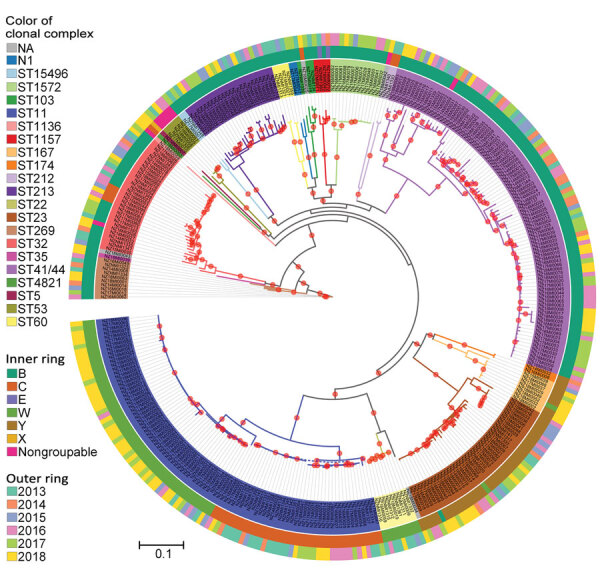
Phylogenetic analysis of New Zealand *Neisseria meningitidis* isolates, 2013–2018. Maximum-likelihood phylogeny was constructed by using a generalized time reversible substitution model and core single-nucleotide polymorphism alignments with RAxML version 8.2.12 (*33*). Branches with >90% bootstrap consensus (200 bootstrap replications) are highlighted with a red dot. Isolate names and clades are colored according to their clonal complex designation. The inner ring indicates the group and outer ring designates the year of isolation of the isolates. N1 lineage corresponds to sequence type that does not have clonal complex designation. NA corresponds to individual isolates where clonal complex is not assigned. Scale bar indicates average number of substitutions per site.

Maximum-likelihood phylogeny constructed from 75,187 core SNP alignments (by using *N. meningitidis* FAM18 as the reference genome) showed 17 highly supported clades (with >90% bootstrap value), 15 of which were consistent with the CC definition (Figure 3). One clade of group B isolates could not be assigned to a CC because its ST had not been assigned a CC designation; it is noted as N1 on the phylogenetic tree. CC11 is the predominant CC consisting of 40 group C and 54 group W isolates; the second most common was CC41/44, consisting of 90 group B and 1 nongroupable isolates.

**Figure 4:**
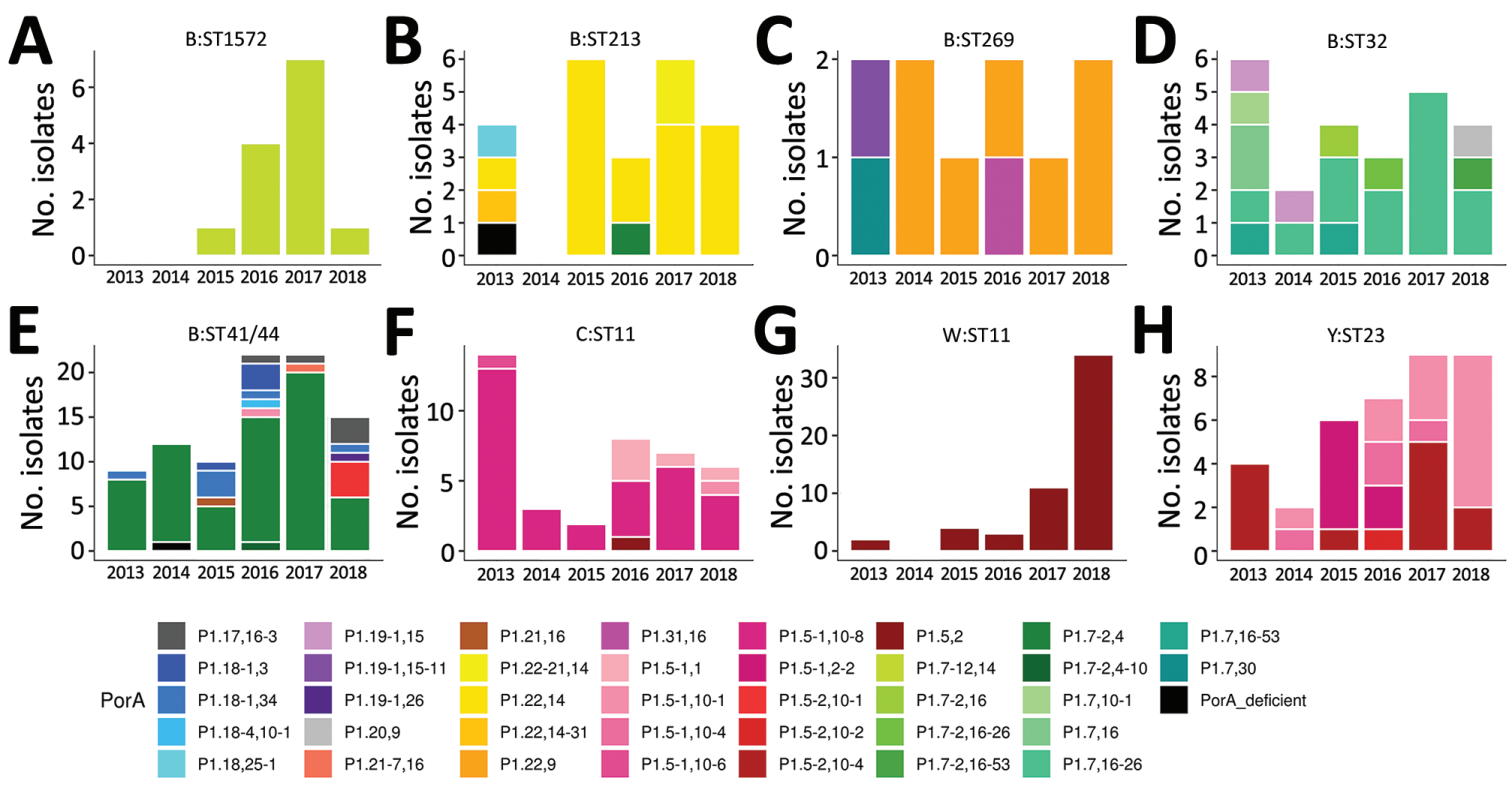
Diversity and prevalence of PorA variable region (VR) variants in common *Neisseria meningitidis* strains in New Zealand, 2013–2018. PorA VR1 and VR2 variant diversity and numbers of common strains are depicted. Strain is defined by group and clonal complex. Only strains with >10 isolates were analyzed.

Since the NZMenB epidemic, New Zealand has used a combination of PorA subtype with group information to define *N. meningitidis* strains. We integrated the PorA and CC information to identify the strains currently circulating in New Zealand (Figure 4).

### Phylogenetic Analysis of New Zealand W:CC11 Isolates

Rates of group W disease in 2018 were higher than in 2013. Most sequenced group W isolates in 2018 (30/35) belonged to CC11. To understand how New Zealand W:CC11 isolates relate to the global group W lineages, we analyzed New Zealand W:CC11 in the context of major W:CC11 lineages. We included 196 CC11 isolates in this analysis (151 downloaded from public databases and 45 invasive isolates from New Zealand). The mapping rate was 92.4%–99.3% when *N. meningitidis* FAM18 (AM421808.1), a representative of CC11, was used as a reference. The mean genome depth was 152X, with 84.8%–97.7% of loci covered at >20-fold (Appendix 1 Table 2).

**Figure 5:**
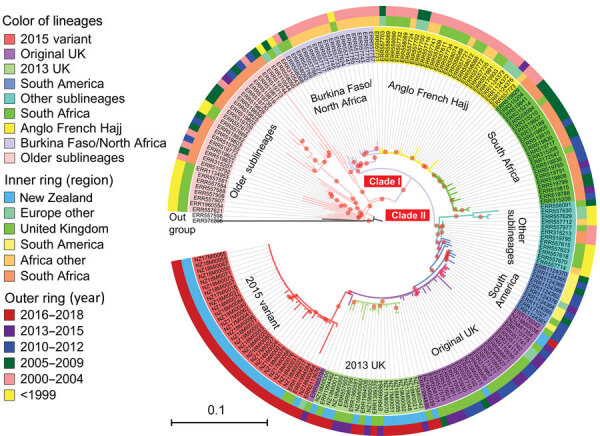
Phylogenetic position of New Zealand group W clonal complex 11 (W:CC11) *Neisseria meningitidis* isolates within the global W:CC11 major lineages. Maximum-likelihood phylogeny was generated by RAxML version 8.2.12 (*33*) on the basis of the core single-nucleotide polymorphism alignment of 198 W:CC11 isolates. Branches with a bootstrap (200 replications) value >90% are indicated with a red dot. Excluding the basal older sublineages, all other isolates form 2 strongly supported clades marked as clade I and clade II, which correspond to the Hajj strain sublineage and the South America strain sublineage. All the major defined lineages of W:CC11 are marked and indicated by consistent background color of isolate’s identification number and branches. The inner ring and outer ring designate the region and year of isolation for each isolate. Scale bar indicates average number of substitutions per site.

We used core SNP (48,507 bps) alignment to construct the phylogenetic relationship of New Zealand W:CC11 isolates within the global W:CC11 (Figure 5). Phylogeny was rooted with 2 group C CC11 isolates. An unrooted neighbor-net phylogeny from the same dataset is shown in Appendix 2 Figure 1. Recombination-corrected phylogeny from the same dataset is shown in Appendix 2 Figure 2. Excluding the basal older sublineages, all other isolates formed 2 strongly supported clades. Clade I corresponds to the previously identified Hajj sublineage, and clade II corresponds to the previously identified South America sublineage. All New Zealand CC11 isolates were located within clade II: 4 clustered with the original UK strains, 12 clustered with the 2013 UK strain, and the other 29 formed a separate cluster with the 2 isolates from the United Kingdom and 1 isolate from Ireland (Figure 5).

To determine whether other isolates are closely related to the new New Zealand cluster, we searched PubMLST and found 27 additional isolates within 50 allele (the cgMLST 0.1 scheme) differences to NZ17MI0022. Because only assembled contigs were available for these isolates, we used a core-genome alignment approach to analyze their relationship within clade II of the W:CC11 isolates. All additional 27 W:CC11 isolates clustered with the New Zealand cluster with high bootstrap support (Appendix 2 Figure 3). The 27 isolates are from Canada and 6 countries in Europe (Appendix 1 Table 3).

Both phylogenetic analyses suggest that the new New Zealand cluster is part of the W:CC11 South America strain sublineage derived from the original UK strain. Because the earliest isolate of the new variant was identified in 2015, we named it the 2015 strain of the W:CC11 South America strain sublineage (the 2015 strain).

### Epidemiology of the 2015 Strain

Similar to the 2013 UK strain, the 2015 strain is associated with higher death rates. During 2017–2018, the 2015 strain was associated with a death rate of 17.8% (6/34 cases) in New Zealand, higher than the rate of 5.9% (10/170 cases) for other groups in the same period (p = 0.03 by Fisher exact test). The 2015 strain also disproportionally affected older adults; 26% of total 2015-strain cases affected adults >60 years of age (9/34), whereas 5.8% (7/121) of total group B cases affected adults in that age group (p = 0.01466 by Fisher exact test).

### 2015 Strain and Penicillin Susceptibility

In 2016, Mowlaboccus et al. (*41*) described a W:CC11 variant circulating in Australia that demonstrated intermediate resistance or was resistant to penicillin and had penA allele 253. To examine whether the New Zealand 2015-strain isolates were also resistant to penicillin, we tested the antimicrobial susceptibility of 42 invasive New Zealand W:CC11 isolates in this study. All 42 isolates were susceptible to ciprofloxacin (MIC <0.03 mg/L), ceftriaxone (<0.12 mg/L), and rifampin (<0.5 mg/L) (Appendix 1 Table 8). We observed variation in penicillin susceptibility among the 42 isolates (Appendix 1 Table 8) by using Clinical and Laboratory Standards Institute breakpoints. Of the 15 isolates belonging to either the original UK strain or the 2013 UK strain, all were susceptible to penicillin (<0.06 mg/L). Of the 27 isolates belonging to the new 2015 strain, 12 displayed intermediate resistance (0.12–0.25 mg/L) and 15 were resistant (>0.5 mg/L). The 2015-strain isolates were significantly more resistant to penicillin (p<0.00001 by Fisher exact test). All 27 New Zealand 2015-strain isolates had penA allele 253, the same allele described in the Australia study (*41*). In the larger dataset that included 30 international 2015-strain isolates, all but 1 isolate had penA allele 253 (Appendix 1 Table 3).

## Discussion

We comprehensively analyzed *N. meningitidis* in New Zealand during 2013–2018 to describe its population structure after the NZMenB epidemic. We examined the rate of IMD and clonal distribution of circulating isolates. We also offer evidence that a distinct variant of W:CC11 is circulating globally and has been causing IMD since 2015.

Meningococcal disease continues to substantially affect the health of persons in New Zealand. In comparison with other developed countries (such as the United States, the United Kingdom, and the Netherlands, which have reported ≤1 case/100,000 population) (*42*–*45*), New Zealand still has a high rate of IMD notification. Although the rate of meningococcal disease in 2018 (2.5 cases/100,000 population) was substantially lower than the peak rate observed during the epidemic (17.4 cases/100,000 population in 2001), rates of IMD in New Zealand have increased since 2014 (Appendix 2 Figure 1).

The distribution of group Y disease and group W disease has changed over the past decade. Incidence of group Y disease has been increasing since the late 1990s in the Americas and since 2010 in Europe (*46*,*47*). The number and proportion of group Y disease in New Zealand began to increase in 2013 (2/85 [2%] in 2012 to 4/68 [6%] in 2013). The increase in group W:CC11 disease here was not significant until 2017 and 2018, when the number of group W cases more than tripled, from 10 to 33. In the United Kingdom, the original UK strain emerged in 2009; the descendant 2013 strain emerged in 2013 and expanded to other countries thereafter (*15*). These data suggest that IMD trends in New Zealand follow global trends with some delay, possibly because of geographic isolation. Therefore, for IMD monitoring in New Zealand, continued detailed typing of meningococcal isolates is critical for obtaining comparable data for participation in global meningococcal surveillance.

By using group and PorA type for strain definition, the diversity index for 2013–2018 period meningococci is slightly higher (Shannon–Wiener index 2.98) than that for 2008–2012, the 5-year period following the end of the epidemic (Shannon–Wiener index 2.81; data not shown). Since 2012, nonepidemic group B cases have regularly surpassed epidemic cases; 47% of group B cases were caused by nonepidemic strains during 2008–2011, and 63% were caused by nonepidemic strains during 2012–2018. The top 3 strains circulating within the nonepidemic group B cases were B:P1.22,14:F5–5:CC213 (13 isolates), B:P1.7,16–26:F3–3:CC32 (13 isolates), and B:P1.7–12,14:F1–7:CC1572 (12 isolates). All 3 strains are present in PubMLST. B:P1.22,14:CC213 is a common strain, however, only 2 isolates contain the FetA-VR:5–5 allele. PubMLST has 41 B: P1.7,16–26:F3–3:CC32 isolates and 6 B:P1.7–12,14:F1–7:CC1572 isolates (accessed November 3, 2020). Taken together, these data suggest that the meningococcal population in New Zealand has become more diverse after the group B epidemic. Comparison of New Zealand with other countries is challenging because no comprehensive public database exists that contains all IMD cases with fine-typing information. The United Kingdom, however, has been depositing most of their IMD typing information into PubMLST since July 2010, and for 2013–2017, the dataset contains 2,790 records and 649 unique strains (group:PorA-VR1,PorA-VR2:FetA:CC, Shannon–Wiener index 4.45). This information suggests that the isolates that cause IMD are less diverse in New Zealand, which may reflect its smaller and more distributed population structure.

The clonal expansion of a new penicillin-resistant clade of W:CC11 was first identified in Australia in 2016 (*41*). Four isolates (PubMLST identification nos. 41966, 42206, 42409, 50313) that were found to be closely related to the Australia clade (cluster B) (*42*) are part of the 2015-strain cluster (Appendix 2 Figure 3), suggesting that the Western Australia clone belongs in the 2015-strain cluster. In our study, the New Zealand 2015-strain isolates were significantly less susceptible to penicillin compared with New Zealand isolates belonging to the original UK or the 2013 UK strain (Appendix 1 Table 8). The penA 253 allele was hypothesized to play a role in decreasing penicillin susceptibility in the new clade of W:CC11 found in Australia (*41*). All isolates in the 2015 variant cluster, except ERR1994517 from the United Kingdom, have the penA 253 allele (Appendix 1 Table 3), suggesting that penicillin resistance is a common characteristic of the 2015 strain. The presence of the penA 253 allele has consequences for the choice of antimicrobial drug to treat IMD. At the end of 2018, a high rate of W:CC11 disease in the Northland area of New Zealand triggered a local vaccination campaign against the disease. Most of these cases were caused by the 2015 strain. Partly because of the increased penicillin resistance of the 2015 strain, in 2018 the New Zealand Ministry of Health recommended ceftriaxone as the first-choice antimicrobial drug for patients with suspected meningococcal disease (*48*).

W:CC11 meningococci are emerging as a diversifying lineage with several new strains occurring in different geographic locations (*10*,*11*,*41*). Phylogenetic analyses suggest that the 2015 strain is part of the major South America–UK (clade II) lineage (Figure 3; Appendix 2 Figure 3), most likely representing a clonal expansion from a single variant within the original UK strain. The increase in New Zealand group W cases is mainly attributable to the 2015 strain identified in this study. In contrast, the 2013 UK strain was largely responsible for the increases seen in Europe (*15*). We found the 2013 UK strain circulating in New Zealand but in low numbers (3/10 cases in 2017 and 6/33 cases in 2018). For reasons unknown, the 2015 strain, rather than the 2013 UK strain, is expanding in New Zealand. Invasive disease provides no selective advantage for meningococcal bacteria, and a strain must remain in carriage in order to expand in a population. The 2015 strain might have originated in the Australia or New Zealand region and is therefore better adapted to the population, either because of more favorable host immunity or climate and living conditions. Its carriage may have precluded expansion of the 2013 UK strain.

Genomic surveillance of *N. meningitidis* has revealed in great detail the genetic diversity and population structure of circulating meningococci in New Zealand; this more refined surveillance enables the tracking of specific strains that are identifiable only by high-resolution phylogenetic analysis. By using this approach, we identified a distinct globally circulating W:CC11 strain, which would not have been possible without genomic information. Our results emphasize the value of obtaining complete genetic information for invasive pathogens and resolving their global evolutionary relationship for identifying the origin and expansion of new pathogenic variants or strains. Such information will benefit surveillance and can be used to help prevent and control future epidemics.

Appendix 1 Additional data for genomic surveillance of a globally circulating distinct group W clonal complex 11 meningococcal variant, New Zealand, 2013–2018.

Appendix 2 More information about genomic surveillance of a globally circulating distinct group W clonal complex 11 meningococcal variant, New Zealand, 2013–2018.
