# Supplementary material for: Genomic Surveillance of a Globally Circulating Distinct Group W Clonal Complex 11 Meningococcal Variant, New Zealand, 2013–2018
Source: Emerg Infect Dis. 2021 Apr;27(4):1087–97. doi: 10.3201/eid2704.191716 (PMC8007299; doi:10.3201/eid2704.191716)
Supplement: Appendix 2 — More information about genomic surveillance of a globally circulating distinct group W clonal complex 11 meningococcal variant, New Zealand, 2013–2018. [file 19-1716-Techapp-s2.pdf]

Article DOI: https://doi.org/10.3201/eid2704.191716

Genomic Surveillance of a Globally Circulating Distinct Group W Clonal Complex 11 Meningococcal Variant, New Zealand, 2013–2018

Appendix 2

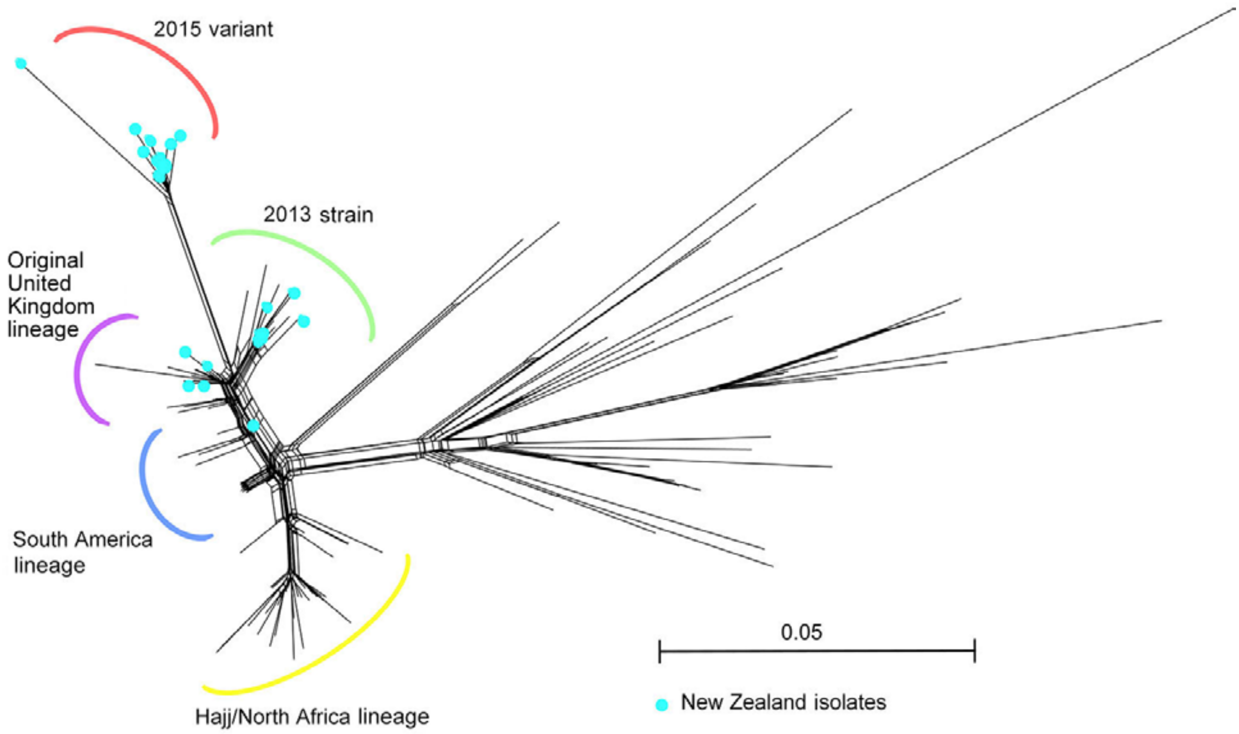

Appendix 2 Figure 1. Neighbor-net phylogeny of New Zealand W:CC11 isolates within the global W:CC11 major lineages. An unrooted neighbor net phylogeny was constructed with SplitsTree version 4 on the basis of the core single-nucleotide polymorphism alignment of 198 W:CC11 isolates. New Zealand isolates are indicated with a cyan dot on the tip of their branch. Major lineages are highlighted. Scale bar indicates average number of substitutions per site.

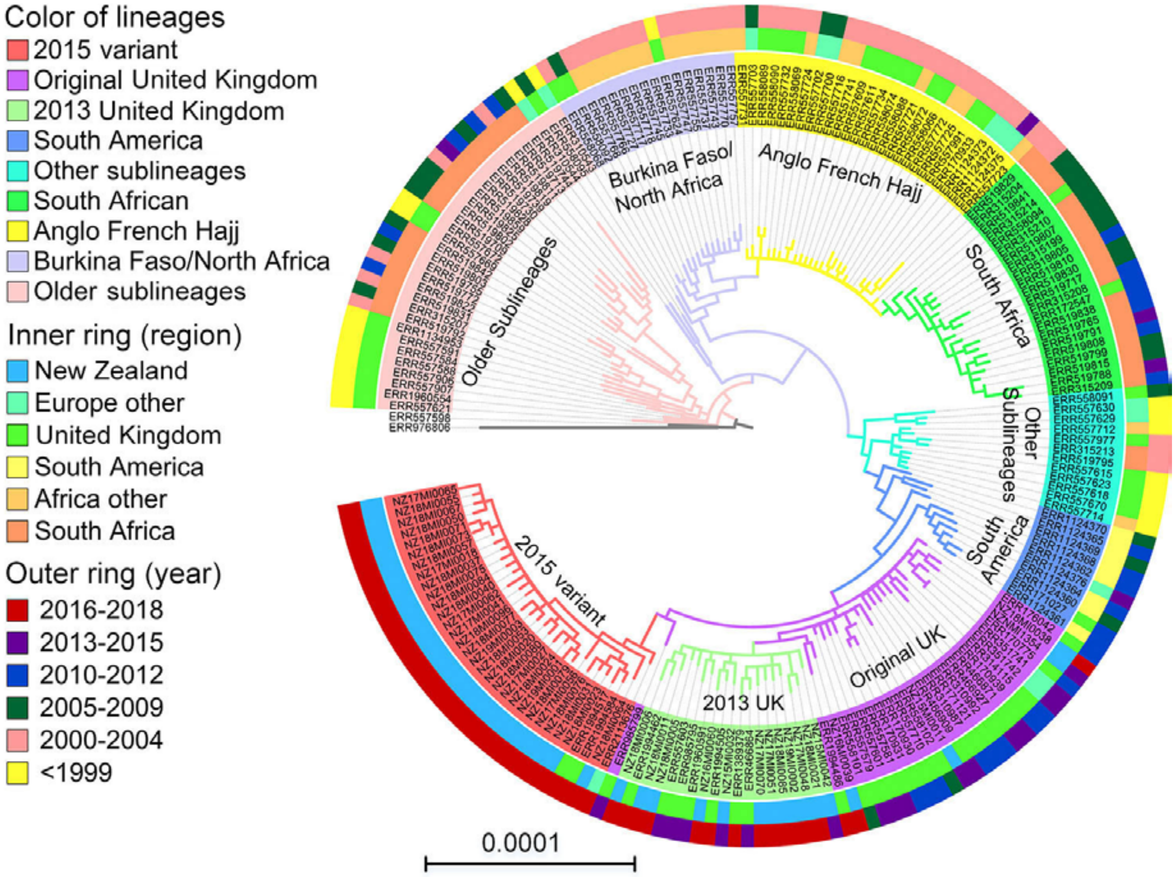

Appendix 2 Figure 2. Recombination-corrected W:CC11 phylogeny. Phylogenetic tree in Figure 5 (https://wwwnc.cdc.gov/EID/article/27/4/19-1716-F5.htm) was corrected for recombination by using ClonalFrameML. Scale bar indicates average number of substitutions per site.

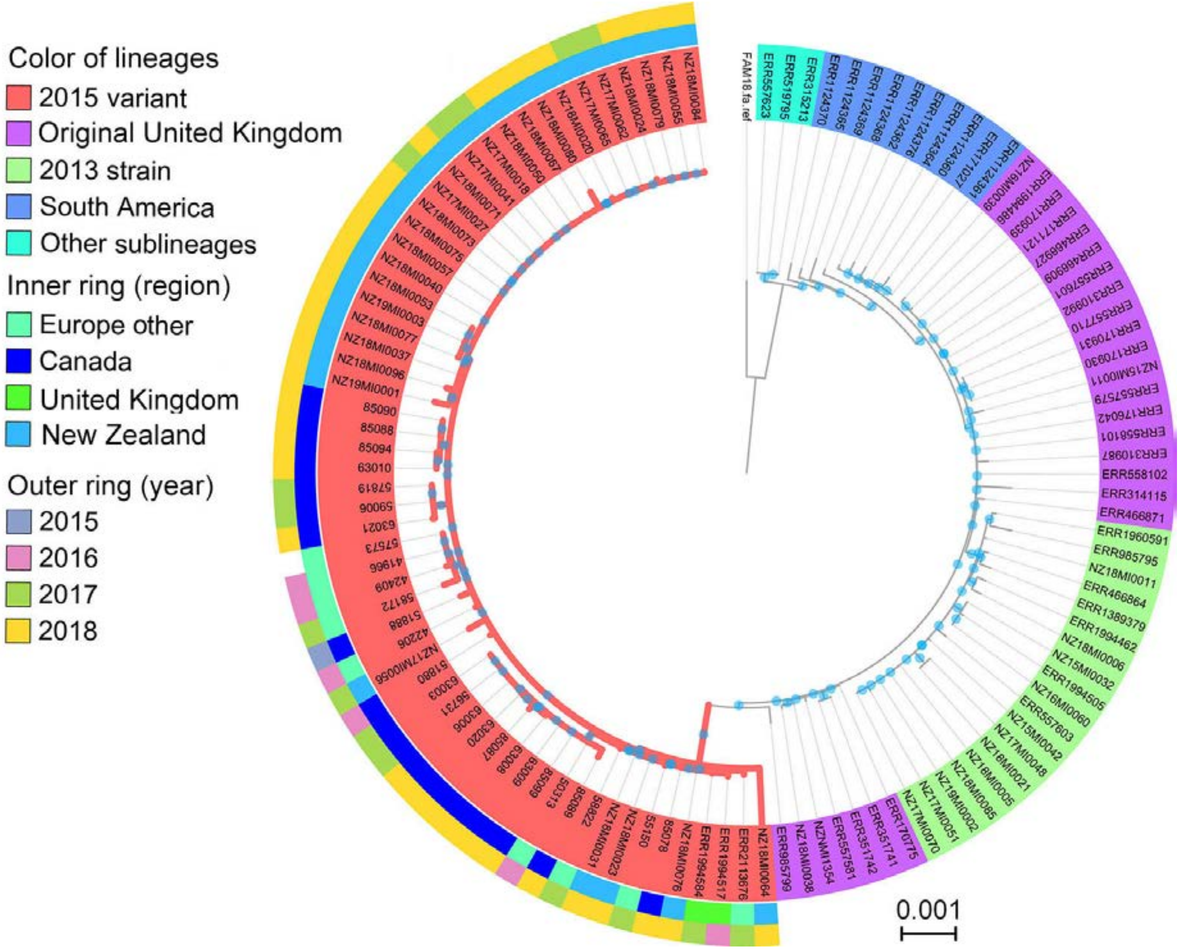

Appendix 2 Figure 3. Circulation of 2015 strain in other countries. A circular phylogenetic tree was generated by RAxML on the basis of the core genome alignment of 118 isolates of the South America–United Kingdom clade. The inner ring and outer ring designate the country and year of each isolate. Branches with bootstrap (200 replications) value >90% are indicated with a blue dot. Scale bar indicates average number of substitutions per site.
